# Correlation Analysis of Anti-Cardiolipin Antibody/D Dimer/C-Reactive Protein and Coronary Artery Lesions/Multiple-Organ Damage in Children With Kawasaki Disease

**DOI:** 10.3389/fped.2021.704929

**Published:** 2021-09-30

**Authors:** Yun-ming Xu, Yan-qiu Chu, Hong Wang

**Affiliations:** Pediatric Department of Shengjing Hospital, China Medical University, Shenyang, China

**Keywords:** anticardiolipin antibody (ACA), D dimer, C reactive protein (CRP), coronary artery lesions (CALs), multiple organ damage, Kawasaki disease (KD), children

## Abstract

**Aim:** Kawasaki disease (KD) is a systemic vasculitis with unknown etiology. In addition to cardiovascular system involvement, it can also have other multiple organs involved. This study is aimed at investigating the correlation between anti-cardiolipin antibody (ACA)/D dimer/C reactive protein (CRP) and coronary artery lesions (CAL)/multiple-organ lesions in children with KD.

**Methods:** Retrospective analysis was performed in 284 KD/IKD patients from May 2015 to April 2016. Among them, 175 were males (61.6%), with average age of 2 years and 5 months old. Patients were divided into ACA+ group and ACA- group, elevated D dimer group (DDE) and normal D dimer group (DDN), and coronary artery injury (CAL) group and non-coronary artery injury (NCAL) group.

**Results:** ACA was most likely tested positive in younger KD children (*p* < 0.05). ACA+ and hypoproteinemia were correlated with CAL, thrombocytosis, and granulocytopenia (*p* < 0.05–0.01). Levels of cTnI and CK in the CAL group were significantly higher than those in the NCAL group (*p* < 0.05). CAL was more frequently detected in younger patients and patients with prolonged fever, later IVIG treatment, and elevated CRP over 100 mg/l, but there was no statistically significant difference (all *p* > 0.05). In the KD with DDE group, the incidence of granulopenia, thrombocytosis, myocardial damage, cholestasis, hypoproteinemia, and aseptic urethritis was significantly higher than that in the KD with DDN group (*p* < 0.05–0.01). However, elevated D dimer was not associated with CAL. CRP elevation was highly correlated with D dimer, but not with CAL.

**Conclusion:** Higher incidence of CAL and myocardial damage occurred in KD patients with positive ACA and hypoproteinemia. In the current study, ACA was only tested for positive and negative, which is a limitation to this study. To further elucidate the association, ACA titers would establish its significance in drawing a conclusion for the significance of ACA in CAL and myocardial damages. In addition, higher incidence of CAL occurred in younger patients. The higher D dimer was associated with increased multiple-organ damage (MOD). CRP was closely correlated with D dimer, but not correlated with ACA and CAL.

## Introduction

Kawasaki disease (KD) is a systemic vasculitis with unknown etiology. It is one of the common connective tissue diseases in children. Currently, KD has emerged as a major pediatric disorder throughout the developed world (1). In developing countries, KD is currently being diagnosed and reported in both China and India (2–4). Multiple organs/systems can be involved in KD. Coronary artery aneurysm (CAA) is the most significant complication that affects the quality of life in the long term. Even in patients receiving IVIG treatment within 10 days of disease onset, the incidence of CAL is still about 5% (5). Prediction of the occurrence of CAL and MOD by utilizing laboratory examination has become a priority for pediatric cardiologists.

In 2014, four KD children with fever but without identified underlying pathogenesis were hospitalized at the department of rheumatism. Results of the ANA\ANCA\ANA titer test, cytokine test (interferon + interleukin), and anticardiolipin antibody (ACA) test indicated that all the four patients had elevated interleukin and two of them were tested positive for ACA and negative for other tests (6, 7). Meanwhile, two KD patients with KD shock syndrome (KDSS) and macrophage activation syndrome (MAS) were transferred to a pediatric intensive care unit (PICU) where the D dimer test was performed (8). Both had significantly elevated D dimer. These clinical aspects prompted us to review literature on ACA and D dimer. As reported in literature, ACA is positive in infection (9), myocardial infarction (10), infectious endocarditis (11), recurrent abortion (12), KD complications (13), systemic lupus erythematosus (14), and stroke (15). Elevated D dimer is associated with vascular endothelial damage (16), and it has been a supporting tool in early diagnosis of KD (17). Furthermore, it is correlated with CAL (18) and so on. We hypothesized that, since KD in children is systemic vasculitis with a high risk in blood clot, ACA+ and D dimer may be associated with disease progression and be used as indications in KD-related complications. Therefore, we added the ACA/D dimer test to the newly admitted children with KD/IKD.

## Methods

### Patients

A total of 284 IKD and KD cases were collected from the pediatric cardiovascular ward of Shengjing Hospital, China Medical University, from May 2015 to April 2016. Among them, there were 175 males (61.62%). The average age of patients was 2 years and 5 months (2 months to 11 years) old. All data were retrospectively analyzed.

### Multiple-Organ Injuries

When two or more than two organs are impacted in the same child with KD around the acute and/or subacute stages.

### Inclusion Criteria

Patients met diagnostic criteria of KD and IKD (19). The diagnostic criteria for CAL were based on the guidelines of the Japanese Circulator Association (20).

### Exclusion Criteria

(1) Incomplete clinical data records; (2) Without ACA and D-dimer tests; and (3) Without IVIG treatment.

### Data Collection

All children diagnosed with KD/IKD had routine tests done at admission: blood routine, liver function, CK, CKMB, hs-cTnT, cTnI, NT pro-BNP, and urine routine. The ACA-IgG antibody (ACA antibody tests were done in 2015) was measured before IVIG in 2 ml of blood using enzyme-linked immunoadsorption assay (ELISA), following the manufacturer's instruction (Beijing Beier Company), and the test is qualitative (the cutoff is 0.1+ negative control OD value, ACA IgG positive ≥ 0.1+ negative control OD value). D dimer was measured in blood with EDTA anticoagulant using immunoturbidimetry for the routine disseminated intravascular coagulation (DIC) test following instructions (Instrumentation Laboratory Company), and the normal range is <252 μg/l. CRP was measured in blood using the radical immunodiffusion method following the manufacturer's instructions (Beckman Coulter, Inc.). Albumin was tested following the manufacturer's instruction (Abbott's Diagnosis, Inc.), using the bromocresol green (BCG) method. CRP/D dimer and albumin were measured several times within 2 weeks of onset. The highest value of CRP/D dimer and the lowest value of albumin were included in the table for statistical analysis.

### Groups

(1) Based on ACA-IgG results, 284 KD/IKD patients were divided into ACA-positive (ACA+) and ACA-negative (ACA–) groups. (2) Based on D dimer results, 280 KD/IKD (4 children without DIC analysis were excluded) were divided into D dimer-elevated (DDE) and D dimer normal (DDN) groups. (3) According to ECHO results, 284 KD/IKD were divided into coronary artery injury (CAL) and no-coronary artery injury (NO-CAL) groups.

### Statistical Analysis

SPSS 22.0 statistical software was used for statistical analysis. The *t* test was used for data in normal distribution. Statistical differences were measured by t test. Median (M) or quaternary interval (p25–p75) was used for data in non-normal distribution. Enumeration data were shown as rate (%), and the chi-square test was used for comparison. The ROC curve was drawn to analyze the predictive value of relevant laboratory indicators on coronary artery injury in KD. *P* < 0.05 indicates statistically significant difference.

## Results

### General Information

**Figure 1:**
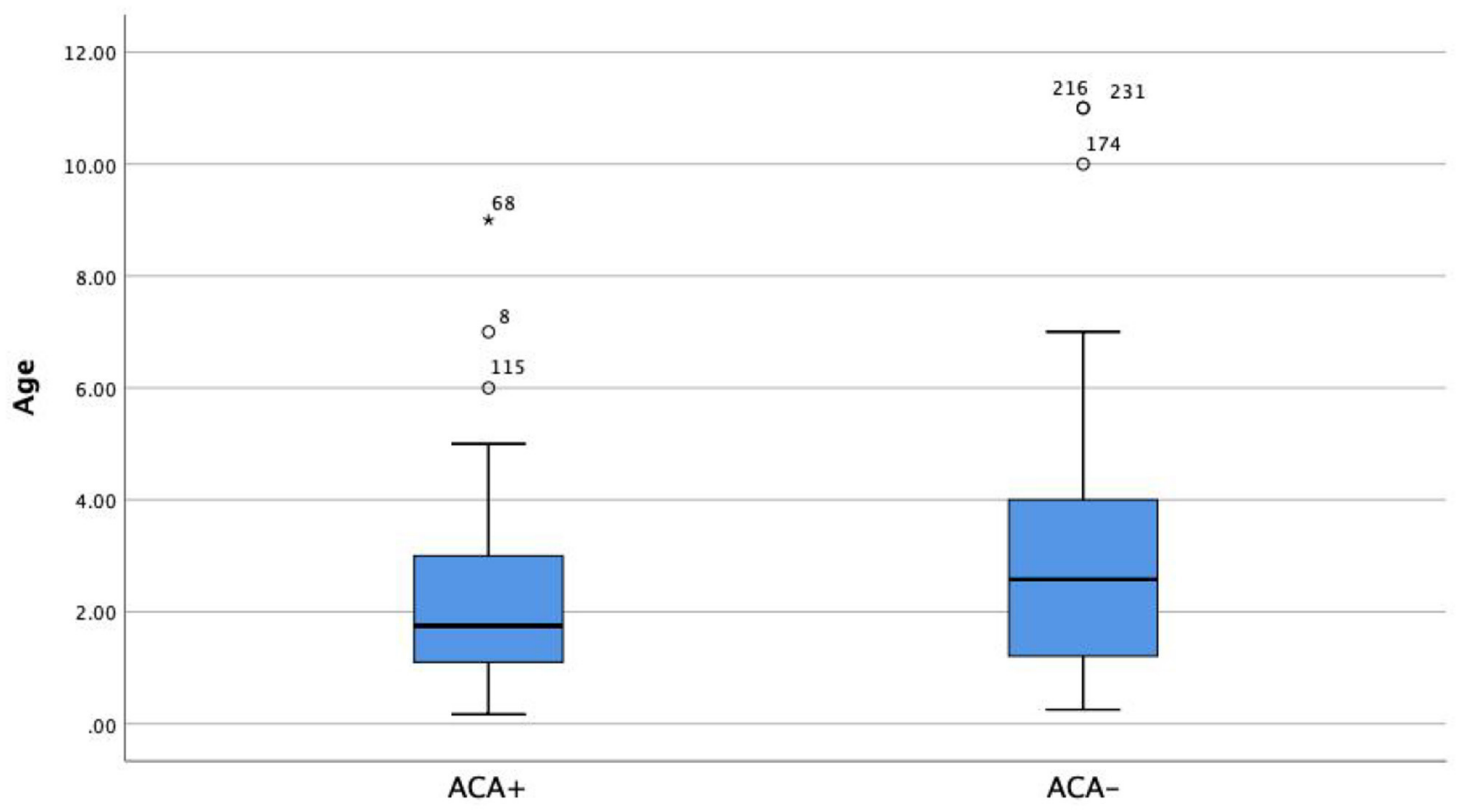
Age difference for ACA+ group vs. ACA– group.

Patients in the ACA+ group were significantly younger (2.1 years old) than those in the ACA- group (2.8 years old) (Figure 1, *Z* = −2.516, *p* = 0.002).

The average time of the first IVIG treatment in the DDE group was 1 day earlier than that in the DDN group (both groups had fever≥ 5 days) (*p* < 0.05). CRP was highly associated with D dimer despite the cutoff value of CRP (all *p* < 0.001), There was no significant correlation between D dimer and ACA (Table 1).

**Table 1:** General information.

**Groups**	***n***	***M* (%)**	**Age (Y)**	**Fever (d)**	**First IVIG (d)**	**IVIG -R (%)**	**CRP ≥ 100 mg/L (%)**	**CRP ≥ 80 mg/L (%)**	**CRP ≥ 50 mg/L (%)**	**CRP ≥ 30 mg/L (%)**
ACA+	168	105(62.5)	2.1 (1.0, 2.9)	8.1	8.0	10	38 (22.6)	52 (31.0)	95 (56.5)	124 (73.8)
ACA–	116	70 (60.3)	2.8 (1.1, 4.0)	8.0	8.0	12	33 (28.4)	48 (41.4)	70 (60.3)	85 (73.3)
*P*		0.714	0.002	0.763	0.581	0.173	0.265	0.071	0.524	0.920
DDE	192	113 (58.9)	1.8 (1.2, 3.0)	8 (7, 9)	7 (6, 8)	14 (7.3)	57 (29.7)	82 (42.7)	135 (70.3)	165 (85.9)
DDN	88	59 (67.0)	2.4 (1.2, 4.0)	8 (6, 9)	8 (6, 10)	7 (8.0)	14 (15.9)	18 (20.5)	30 (34.1)	44 (50.0)
*P*		0.191	0.222	0.274	0.002	0.845	0.014	0.000	0.000	0.000
CAL	17	11 (64.7)	2.1 ± 1.3	10.2 ± 5.2	9.2 ± 4.4	3 (17.6)	7 (41.18)	7 (41.18)	10 (58.82)	14 (82.35)
NO-CAL	267	164 (61.4)	2.4 ± 1.8	8.0 ± 2.3	7.7 ± 2.0	19 (7.1)	66 (24.72)	93 (25.34)	154 (57.68)	190 (51.77)
*P*		0.787	0.433	0.114	0.186	0.134	0.153	0.595	0.926	0.413

*DDE, D-dimer elevated; DDN, D-dimer normal; IVIG-R, IVIG resistance; CRP, C reactive protein.*

### The Correlation Between ACA/D-Dimer and Myocardial/Liver Damage in KD/IKD Children

There were no significant differences in myocardial and liver damages between CAL and NO-CAL groups (*p* > 0.05).

Based on the reported average of AAR at 1.1 by Wang et al. (21), 10 patients in the CAL group had lower AAR (*p* = 0.855).

**Figure 2:**
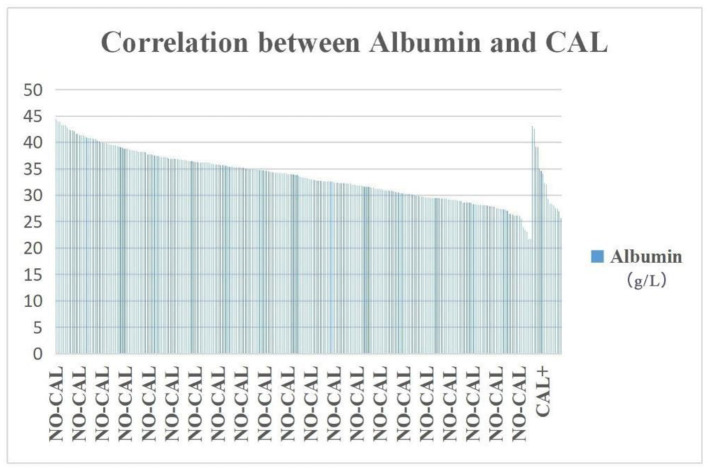
The incidence of hypoproteinemia was significantly higher in the CAL group than in the NO-CAL group (*p* < 0.05). The X-axis indicates with or without presence of CAL. The Y-axis represents the albumin (g/L).

NT pro-BNP, ALT, AST, and TBA were significantly higher in the DDE group than those in the DDN group (*p* = 0.002, 0.035, and 0.002, respectively), whereas ALB and AAR were significantly lower in the DDE group than in DDN (*p* = 0.006 and 0.000, respectively) (Table 2). The incidence of hypoproteinemia was significantly higher in the CAL group than in the NO-CAL group (*p* < 0.05) (Figure 2).

**Table 2:** The correlation between ACA/D-dimer and myocardial/liver damage in KD/IKD children.

**Groups**	***n***	**CKMB** **(>24U/L)**	**CTNI** **(>0.04 μg/L)**	**Hs-CTNT** **(>0.014 ng/mL)**	**NT-pro BNP** **(>300 pg/mL)**	**TBA** **(> 10 umol/L)**	**ALT** **(>40 U/L)**	**AST** **(>35 U/L)**	**ALB** **(<30 g/L)**	**AAR**
ACA+	168	20 (17, 27)	0.005 (0, 0.011)	0.005 (0, 0.007)	358 (169,1049)	5.7 (2.9,9.9)	20 (11.7,43.5)	25 (20,37)	33.5 (29.9,36.3)	1.42 (0.68,1.91)
ACA–	116	20 (17, 25)	0.009 (0, 0.003)	0.005 (0,0.007)	500 (150,1378)	6.3 (3.8,12.1)	17.5 (11,65)	24 (17,34.8)	33 (29.5,37.2)	1.41 (0.77,2.01)
*P*		0.556	0.137	0.291	0.152	0.698	0.815	0.061	0.771	0.961
*DDE*	192	(190) 20 (17, 24)	(187) 0.010 (0.010, 0.012)	(188) 0.005 (0.003, 0.007)	(188) 543.6 (228.5, 1377)	(169) 6.4 (3.4,11.3)	20 (11, 66.5)	25.5 (19, 35.5)	32.3 (29.3, 35.7)	1.24 (0.58, 1.91)
*DDN*	88	(87) 22 (17.5, 28)	(86) 0.010 (0.010, 0.010)	(85) 0.004 (0.003, 0.007)	(84) 185.4 (66.2, 484.7)	(80) 4.9 (3.2, 8.4)	16 (11, 23)	23 (18, 34)	36.2 (33.1, 39.1)	1.61 (1.15, 2.08)
*P*		0.160	0.002	0.145	0.000	0.002	0.002	0.035	0.000	0.006
CAL	17	17 (15.5, 21.5)	0.01 (0, 0.024)	0.004 (0, 0.007)	358 (130, 1323)	3 (23.1)	5 (31.3)	3 (18.8)	32.6 (28.0,35.1)	1.41 (0.78,1.68)
NO-CAL	267	20 (17, 26)	0 (0, 0.01)	0.005 (0, 0.007)	435.5 (153, 1116)	65 (27.8)	72 (27.3)	67 (25.4)	33.9 (30.2,36.9)	1.43 (0.75,2.00)
*P*		0.110	0.013	0.698	0.838	1.000	0.775	0.768	0.364	0.933

*CKMB, creatine kinase isoenzyme MB; CTNI, cardiac troponin I; Hs-CTNT, high sensitive cardiac troponin T; NT-proBNP, N-terminal brain naturetic peptide premise; TBA, total bile acid; ALT, alanine transaminase; AST, aspartate transaminase; DDE, D-dimer elevated; DDN, D-dimer normal; AAR, AST/ALT ratio; the average of AAR is 0.8.*

### The Correlation Between ACA/D Dimer and the Incidence of Multiple-Organ Injuries

The incidence of granulocytopenia, thrombocytosis, and CAL in the ACA+ group was significantly higher than in the ACA- group (*p* < 0.01–0.05).

The incidence of elevated NT pro-BNP and ALT, hypoproteinemia, sterile urethritis, and thrombocytosis was significantly higher in the DDE group than in the DDN group (*p* < 0.01–0.05) (Table 3).

**Table 3:** The correlation between ACA/D-dimer and the incidence of multiple-organ involvement.

**Groups**	***n***	**CAL (%)**	**Granulopenia**	**Anemia (Hb <90 g/l)**	**Thrombocytosis**	**CTnI (>0.04 μg/l)(%)**	**NT pro-BNP**	**ALT (>40 U/l) (%)**	**Hypoproteinemia**	**Cholestasis**	**SM**	**Pneumonia**	**SU**	
			**(N <1.0 ×10^**9**^/l)**		**(PLT > 450 ×10^**9**^/l)**		**(>300 pg/ml)(%)**		**(ALB <30 g/L) (%)**	**(TAS>10 umol/l) (%)**				
ACA+	168	14 (8.3)	75 (44.6)	22 (13.1)	111 (66.1)	2/164 (1.2)	88/164 (53.7)	45 (26.8)	72/168 (42.9)	25/142 (17.6)	10 (6.0)	58 (34.5)	21 (12.5)	
ACA–	116	3 (2.6)	31 (26.7)	13 (11.2)	61 (52.6)	5 (4.3)	71/110 (64.5)	33 (28.4)	32/116 (28.3)	27/108 (27.6)	11 (9.5)	34 (29.3)	20 (17.2)	
*P*		0.045	0.002	0.634	0.022	0.130	0.073	0.758	0.540	0.009	0.264	0.356	0.264	
														ACA+
*DDE*	192	12 (6.3)	53 (27.6)	24 (12.5)	125 (65.1)	6 (3.1)	128/187 (68.4)	63 (32.8)	59 (30.7)	32 (16.7)	16 (8.3)	66 (34.4)	31 (16.1)	113 (59.2)
*DDN*	88	4 (4.5)	19 (21.6)	10 (11.4)	44 (50)	0 (0)	28/83 (33.7)	14 (5.9)	4 (4.5)	11 (12.5)	4 (4.5)	26 (29.5)	2 (2.3)	30 (34.1)
*P*		0.568	0.942	0.787	0.016	0.182	0.000	0.003	0.000	0.732	0.514	0.424	0.004	0.000

*DDE, D-dimer elevated; DDN, D-dimer normal; SM, sterile meningitis; SU, sterile urethritis.*

**Figure 3:**
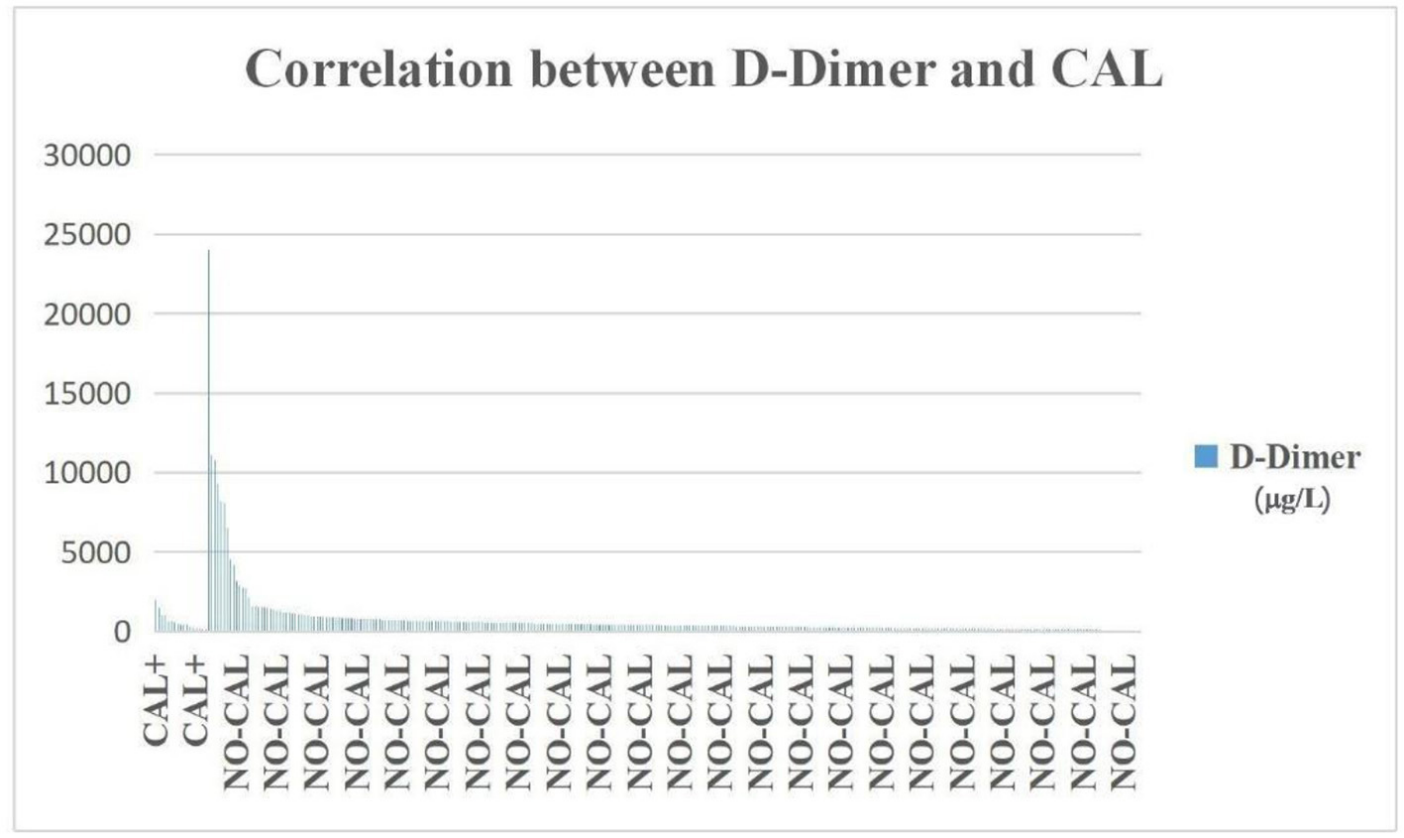
The incidence of D-dimer was not significantly higher in the CAL group than in the NO-CAL group (*p* > 0.05). The X-axis indicates with or without presence of CAL. The Y-axis represents D-dimer in μg/L.

In the DDE group, there were 113 patients who were ACA+ (59.2%), which was significantly higher than in the DDN group (*p* < 0.000), but the incidence of DDE was not significantly higher than DDN in the CAL group (Figure 3).

### The Correlation Between CAL and Myocardial/Liver Damages

CAL group presented with significantly higher incidence of hypoproteinemia and cTnI, (*p* < 0.05). CAL occurred frequently in patients who were younger, with prolonged fever, later IVIG treatment, CRP elevated over 100 mg/L, and lower AAR. However, there were no significant difference (*p* > 0.05; Table 4).

**Table 4:** The correlation between CAL and CRP/D-Dimer/myocardial damage/Hypoproteinemia.

**Groups**	**Gender M (%)**	**Age (years)**	**Fever (days)**	**First IVIG (days)**	**CRP≥100 mg/l (%)**	**D-Dimer (ug/L)**	**CTnI (μg/L)**	**Hs-cTnT (ng/mL)**	**NT-proBNP (pg/mL)**	**Hypoproteinemia (%)**
CAL (*n* = 17)	11 (64.7)	2.1 ± 1.3	10.2 ± 5.2	9.2 ± 4.4	7 (41.2)	622.9 ± 509.3	0.01 (0, 0.024)	0.004 (0, 0.007)	358 (130, 1323)	8 (47.06)
NO-CAL (*n* = 267)	164 (61.4)	2.4 ± 1.8	8.0 ± 2.3	7.7 ± 2.0	64 (24.0)	726.3 ± 1835.3	0 (0, 0.01)	0.005 (0, 0.007)	435.5 (153, 1116)	65 (17.71)
*P*	0.787	0.433	0.114	0.186	0.146	0.823	0.013	0.698	0.838	0.047

### The Receiver Operating Characteristics (ROC) of cTnI Used in Predicting Coronary Artery Damage in Children With KD

**Figure 4:**
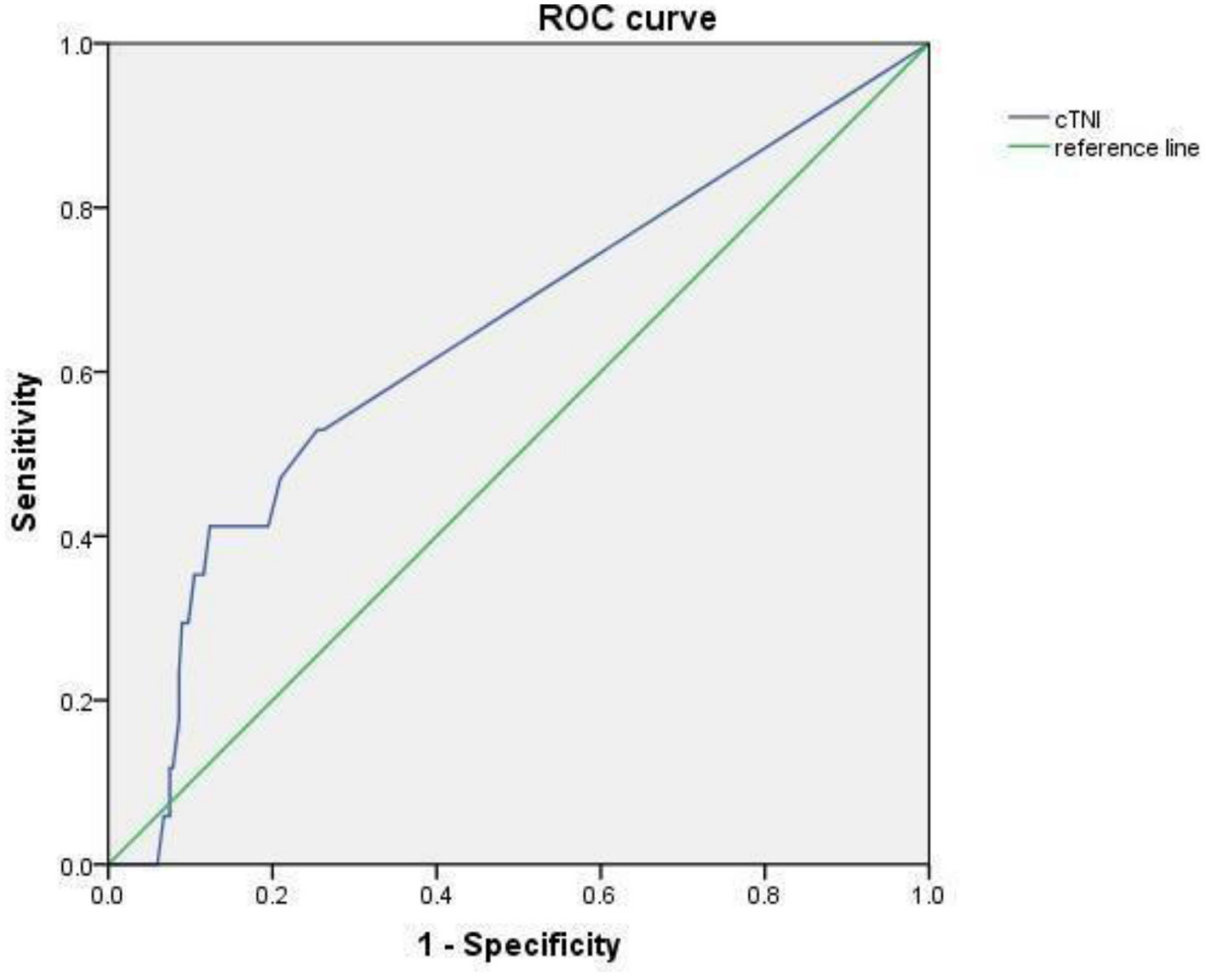
The ROC curve of cTnI levels in predicting CAL in children with KD.

The AUC of the area under cTnI curve was 0.657, 95% CI was 0.508–0.805 (*p* < 0.05). When 0.0175 was used as the cutoff value, the sensitivity and specificity for predicting CAL were 0.412 and 0.876 (Figure 4).

## Discussion

### The Current Clinical Application of ACA Test

ACA are anticardiolipin antibodies. They were first detected using radioimmunoassay (22). Under normal conditions, the immune system does not recognize cardiolipin that lies at the inner mitochondrial membrane. However, once cardiolipin is exposed, it will trigger an immune response and produce the autoimmune antibody ACA. Immunophenotypes include IgG, IgM, and IgA. Among them, IgG is a pathogenic autoantibody, and its high titer and long half-life make it an important player in immune response. In a study including 34 Italian KD children, anticardiolipin (aCL) was detected in 30% of KD patients (IgG aCL antibodies were found in 14 patients, IgM aCL in 1, and 1 had both) (23). The target antigen of ACA is negatively charged cardiolipin on the platelet and endothelial cell membranes. ACA can stimulate immune response to the phospholipids of its own cell membranes and damage the cell membranes and thus release a large amount of inflammatory cytokines and chemokines and further trigger vascular inflammation. ACA is commonly found in the following diseases: (1) thrombotic diseases, such as myocardial infarction (24), stroke (25), and habitual abortion. There is marked activation of the endothelium and immune system in KD. Anticardiolipin antibodies (aCL) can cause activation of the endothelium. An increase in IgA anticardiolipin antibodies in KD patients suggests that the degree of increase in these antibodies correlates with the degree of systemic inflammation (26). Low IgG was not a risk factor for CAAs in this study. However, KD patients with relatively high IgG prior to treatment may have an increased risk of resistance to initial IVIG therapy (27). Studies have suggested that (13, 28) ACA, as one of laboratory indications for thrombosis, can cause damage in vascular endothelial cells, interfere with the coagulation system, affect platelet function, and reduce fibrinolytic activity. Finnazzi et al. (29) performed a blind study on 360 ACA-positive patients and concluded that a high level of ACA was a meaningful indicator for predicting thrombosis. Sueoka et al. (30) raised an alarm in patients who had developed thrombosis in the presence of positive ACA after percutaneous thoracoscopic surgery. There have been discussions on the clinical significance of positive ACA antibodies. In healthy controls, aCL antibodies were found in five patients (22%) (23). In order to differentiate from false-positive interference, many medical institutions have implemented testing ACA antibody titers (31) (our hospital is currently using semi-quantified). (1) ACA is also found in connective tissue diseases, such as systemic lupus erythematosus and KD. (2) ACA can be detected in patients infected with syphilis, AIDS, hepatitis C, tuberculosis, parvovirus, and cytomegalovirus. Thus, there is a limitation in evaluating the association of ACA-IgG with vasculitis in Kawasaki disease. ACA titer may stratify the association between ACA and Kawasaki disease. In our study, the incidence of CAL and the proportion of thrombocytosis in children with elevated ACA were significantly increased, suggesting a possible mechanism that inflammatory mediators and the activation of negatively charged cardiolipin in platelets caused damages in the vascular endothelium, which increased the risk of thrombosis. Our results showed that CAL was more common in ACA+ patients. This is consistent with literature reports (32). In contrast to the correlation between CAL and low AAR reported by Wang et al. (21), our results showed that CAL was not associated with AAR. On the other hand, elevated D dimer was correlated with low AAR and NT pro-BNP, but not with CAL. Since the COVID-19 outbreak, there have been various complications related to thrombosis, and they are accompanied by DIC and an increase in ACA (33, 34). These results suggest that COVID-19 virus can activate vascular endothelium and cause endothelial damage and intravascular coagulation.

### The Significance of Elevated D Dimer

The D dimer test is often used in clinical diagnosis of diffuse intravascular coagulation (DIC), deep vein thrombosis (DVT), pulmonary embolism, myocardial infarction, cerebral infarction, etc. This index can not only be used for the diagnosis of thrombotic diseases but also be used as an index for monitoring the dose of thrombolytic drugs for therapeutic effect. KD itself is an inflammation-mediated vasculitis that activates the vascular endothelium and causes endothelial damage. Symptoms are similar to multisystem inflammatory syndrome in children with COVID-19. Elevated D dimer is associated with DIC hypercoagulation and macrophage activation and aggravates tissue disintegration (32). In addition, elevated D dimer has been reported to be correlated with CAL (35) and complications in multiple organs. Our study showed that elevated DD was only correlated with complications in MDO but not with CAL. Kong et al. (36) reported that elevated D dimer was also correlated with IVIG resistance in KD children (18). However, our results showed that D dimer elevation was not associated with IVIG resistance. We also found that KD with MAS complication and necrotizing pneumonia was not associated with CAL, although patients had fever which lasted for more than 2 weeks. CRP increased surprisingly (7). The conclusion drawn from our study disagrees with other reports in literature (37). In this study, there were two KD children with elevated D dimer over 10,000 μg/L (D dimer at 24,007 μg/l in one 19-month-old boy with pneumonia and aseptic meningitis and at 10,773 μg/l in the other 17-month-old girl with hemophagocytic syndrome, hyponatremia, pneumonia, bilateral pleural effusion, liver function damage, hypoproteinemia, pancreatic injury, and aseptic meningitis). Both patients did not have CAL. It is consistent with the report by Ming-Tsan et al. (38). Data at our center showed that there was no relationship between increased D dimer and IVIG resistance either, which is conflicted with reported studies (39). This may be associated with earlier IVIG treatment by 1 day in DDE, or it may be related to the differential expression of immune responsive genes during the KD occurrence and development (40).

### D Dimer in Children With Multisystem Inflammatory Syndromes

Last year, the coronavirus disease 2019 (COVID-19) hit the world, and a new manifestation emerged as a multisystem inflammatory syndrome in children (MIS-C) which carries similar clinical symptoms of KD, including toxic shock syndrome and severe sepsis (41). The severity is significantly correlated with D dimer, even KDSS (42). COVID-19 linked with thrombotic microangiopathy triggers multiple vasculitis along with arteriole thrombosis, and medium and large venous and arterial vessels mediate the disseminated intravascular coagulation (DIC) (43). Additionally, 52% of MIS-C has elevated D dimer (44). Although some children with KD in our center last year had very similar symptoms to COVID-19 with MIS-C (45), we did not find any evidence of COVID-19 infection in these patients. However, the ultimate treatments for these patients rely on IVIG and glucocorticoid, which are similar to treatments for rheumatic immune diseases such as KD (46).

## Conclusion

Our study indicated that ACA+ and hypoproteinemia were correlated with CAL, granulocytopenia, and thrombocytopenia in KD children. In the current study, ACA was only tested for positive and negative, which is a limitation to this study. To further elucidate the association, ACA titers would establish its significance in drawing a conclusion for the significance of ACA in CAL and myocardial damages. Elevated D dimer was correlated with MOD but not with CAL; CRP was correlated with D dimer, but not with ACA and CAL.

## Data Availability Statement

The raw data supporting the conclusions of this article will be made available by the authors, without undue reservation.

## Author Contributions

YX: patient's observation, data collection and analysis, and the editing of manuscript. YC: patient's observation and data analysis. HW: patient's diagnosis, treatment, data analysis, and the editing of manuscript about discussion. All authors contributed to the article and approved the submitted version.

## Conflict of Interest

The authors declare that the research was conducted in the absence of any commercial or financial relationships that could be construed as a potential conflict of interest.

## Publisher's Note

All claims expressed in this article are solely those of the authors and do not necessarily represent those of their affiliated organizations, or those of the publisher, the editors and the reviewers. Any product that may be evaluated in this article, or claim that may be made by its manufacturer, is not guaranteed or endorsed by the publisher.
